# Allograft bone banking experience in Nigeria: a review of first 2 years

**DOI:** 10.11604/pamj.2024.49.105.41103

**Published:** 2024-12-02

**Authors:** Kehinde Adesola Alatishe, Oluwaseyi Kayode Idowu, Mustapha Alimi

**Affiliations:** 1 Orthopaedic and Trauma Department, National Orthopaedic Hospital, Lagos, Nigeria

**Keywords:** Bone allograft, bone banking, transplant, Nigerian experience

## Abstract

Bone allografts are increasingly being used in various orthopaedic surgeries all over the world but were not readily available in Nigeria until year 2020 when the first bone bank facility was established at our hospital. The paper aimed to share our experience and the challenges faced within the first 2 years of operating the first bone bank in Nigeria. We retrospectively reviewed our experience between 1^st^ September 2020 and 31^st^ August 2022. The donors were selected based on American Association of Tissue Bank (AATB) guidelines and Nigerian National Blood Transfusion policy. Our preference was the use of allograft without late donor testing for HIV seroconversion. However, the allografts were treated, irradiated at 25 kGy, and stored in a freezer at -80°C. A total of 88 bone grafts were retrieved, processed and stored in the bone bank over the 2-year period. All allografts were from living donors. Of these, 55 (62.5%) bones were retrieved from female donors and 33 (37.5%) from males. The mean age of all donors was 55.9±15.34 years (range: 32-90 years). Bone grafts issued out from the bank were 28/88 (31.8%) in all. There was no single case of clinical infection reported. The challenges observed were limited long bone allografts, low patronage among surgeons, lack of institution preparedness to process bones from fresh dead donors and disinterest from some surgeons. Running of the first bone bank facility in Nigeria has been successful thus far. The challenges can be surmounted by creating awareness amongst the populace and surgeons of the availability and safety of bone allografts.

## Introduction

A bone bank is defined as a facility that is engaged in donor screening, retrieval, processing, storage and distribution of bone tissue. Bone allografts are increasingly being used in various orthopaedic surgeries such as complex primary and revision arthroplasties, spine fusion, ligament reconstruction and skeletal reconstruction after tumor excision [1,2]. However, these allografts were not readily available in Nigeria until year 2020 when the first bone bank facility was established at our regional orthopaedic hospital. The running of a bone bank is not without its own challenges which could be mitigated by training and re-training of staffs on the operational modus, careful donor selection by the orthopaedic surgeon, providing constant power supply, maintaining cold chain during storage and transportation of graft within the country. The operations of the bone bank require well-organized quality control mechanisms that help detect errors during procurement, processing, storage and transplantation of allografts by strictly adhering to local and /or international guidelines. One of the dreaded complications of allograft is infection transmission and has been ascribed to poor donor selection, graft handling and processing [3]. Medical history to exclude chronic disease or any specific risk of the donor harboring transmissible diseases cannot be overemphasized. Some activities such as intra-venous drug abuse, recent tattooing, unprotected sexual intercourse and previous blood transfusion are vital in excluding donors. The data from the UK showed that after pre-screening with medical and behavioral history, the risk of a donor testing positive for viral pathogens during the second phase of donor screening was less than 1 in 1,500 [4]. According to the American Association of Tissue Bank (AATB) guidelines, the mandatory minimum donor testing should include serological investigations for Human immunodeficiency virus (HIV) types 1 and 2, hepatitis B and C viruses, syphilis, Human T-cell lymphotropic virus (HTLV) types 1 and 2 [5].

Sources of bone allografts could be from dead or living donors, but most bone banks have reported living donors as their primary source of allografts with femoral heads being the most common [6-8]. Strong emphasis should be laid on ethical considerations of harvesting or transplanting bone allografts in a tissue banking industry. Valid informed consent for bone donation should be obtained from the donors or their next-of-kin. Bone allograft must be prepared, processed and preserved in a manner that minimizes microbial growth and improves the quality of the graft for transplantation [9].

All tissue-cleansing or handling processes must be geared towards further reducing the possibility of disease transmission without causing biological or mechanical harm to the allografts. For instance, terminal sterilization of the bone allograft with gamma irradiation is believed to minimize the risk of infection but the effect on bone biology and biomechanics is still controversial [10]. A good record keeping of data from both donors and recipients is imperative for auditing and reappraisal of activities of tissue banking. This paper aims to highlight our preferred processing technique, share our experience and challenges of operating a bone bank in Nigeria within the first 2 years.

## Methods

**Study setting:** hospital-based study

**Study participants:** patients who had bipolar hip hemiarthroplasty or total hip replacement; whose native femoral head met the criteria for donation into the bone bank.

**Study design:** this was an observational descriptive study of our experience on the procurement, processing, storage and usage of bone allografts at our bone bank facility between 1^st^ September 2020 and 31^st^ August 2022.

**Ethical consideration:** the Nigerian National Health Act of 2014, sections 48-57 have several provisions governing organ or tissue donation and transplantation [11]. Femoral heads retrieved during arthroplasty are considered as operative residues, with informed consent from the patients to preserve, process, store and distribute them for clinical purposes as stated in the consent form of our hospital. Ethical clearance was sought and approval was granted by the ethical committee of the hospital.

**Sample recruitment (allograft procurement):** allografts were obtained from living donors after informed consent. Donor selection was based on American Association of Tissue Bank (AATB) guidelines [5] and Nigerian National Health Act of 2014 [11]. Detailed history, examination and blood screening of donors was carried out to exclude infectious diseases of bacterial or viral origin including syphilis, HIV 1 and 2 and hepatitis B and C. Bones were accepted only from those donors with negative reports for the above bacterial and viral infections. Blood group and rhesus typing were done for all donors. Patients with malignancies or autoimmune disorders were excluded from donating bone.

**Allograft processing:** retrieved bones were processed through mechanical and chemical means. Mechanical debridement of soft tissues was carried out by the operating orthopaedic surgeon with surgical instruments in the modular theatre under aseptic condition. These bones were then immersed and serially cleansed in normal saline; packed and sealed in three- layered poly-ethylene packets; properly indexed and transported to the bone bank in an iced medium within 30 minutes of collection. A duly filled form containing donor screening tests, details about the bone graft, time of collection and informed consent of the donor accompanied the graft to the bone bank.

**Storage:** femoral heads excised during surgery and bones extracted from traumatic amputated limbs were stored in a chamber of an ultra-low temperature freezer at -80°C until gamma irradiation and distribution. Terminal Sterilization: The bone allografts were sterilized by exposure to 25 Gy of gamma rays at a tertiary hospital´s radiotherapy department in close proximity to our center. These allografts were subsequently stored in a separate chamber in the ultra-low freezer.

**Indexing:** the bone grafts were properly labeled and coded; and information regarding the donor and recipient, types of bones, results of investigations, dates of procurement and date of issue were entered in a register maintained at the bone bank.

**Distribution and usage:** the bone allografts were distributed within and outside our hospital for various orthopaedic and trauma uses as structural and/or morselized forms. Cutting the grafts into different sizes and shapes prior to terminal sterilization is still a challenge we are hoping to resolve in a short while. However, the surgeons usually defrost the graft by soaking in povidone iodine or saline solution for about 30 minutes before usage.

**Statistical analysis:** data was collected from the bank registry and analyzed with the Statistical Package for Social Science (SPSS) software version 20 (IBM corp., USA). Descriptive statistics were used to summarize the data and presented in percentages, frequency tables and charts.

## Results

A total of 88 bone grafts were retrieved, processed and stored in the bone bank over the 2-year period. Most donors (97.7%) were living patients who undergone hip hemiarthroplasty or total hip replacement for either neck of femur fractures or severe osteoarthritis respectively. The remaining 2.3% of the donors were living patients who had stump refashioning for traumatic mangled extremity. Of these, 55 (62.5%) bones were retrieved from female donors and 33 (37.5%) from males. The mean age of all donors was 55.9±15.34 years (range: 32-90 years). Types of bone grafts included: femoral heads (82), distal femur (1), proximal tibia (1), fibula (1), tibia diaphysis (1), distal tibia (1) and foot bones (1). The processing technique was as described in the methodology with emphasis on terminal sterilization with gamma irradiation. Bone grafts issued from the bank and transplanted within and outside the hospital were 28/88 (31.8%). These included 26 femoral heads, tibial diaphysis (1) and fibula (1). Among these, 22 grafts were utilized within the hospital while the remaining six grafts were used at other tertiary centers or private hospitals within the country. The allografts were used for acetabular reconstruction in complex primary and revision total hip replacement, proximal femur bone- allograft composite reconstruction in revision THR, non- union, tibial plateau fractures and for spinal fusion as outlined in (Table 1). In a feedback questionnaire sent to all surgeons who used the bone allograft, there was no incidence of clinical surgical site infection and most surgeons were satisfied with the quality of the graft. The challenges observed were limited long bone allografts, low patronage among surgeons; lack of bank preparedness to process bones from fresh dead donors and apathy from some surgeons due to probable infection risk and possible poor osteointegration.

**Table 1:** uses of bone allografts in orthopaedic procedures

Orthopaedic procedures	Numbers N (%)
**Acetabular reconstruction with total hip replacement**	18(64.3)
**Proximal femur bone-allograft composite reconstruction in revision total hip replacement**	1(3.6)
**Spinal fusion**	3(10.7)
**Tibial plateau fractures**	2(7.1)
**Non-union of the femur**	3(10.7)
**Non-union of the tibia**	1(3.6)
**Total**	28(100)

## Discussion

The operation of a bone bank facility in Nigeria is a new development with fast growing demand for processed bone allograft in all parts of the country. This paper aimed to share our experience in running a bone bank; highlighting the challenges and ways of improving our services in the nearest future. The bone bank committee comprises of Orthopaedic surgeons, pathologist, medical microbiologist, laboratory technician, theatre nurses, research assistant and a Clerk. The Head of the committee is a consultant orthopaedic oncologist and also the head of Clinical services of the hospital (second author). While, the day- to-day activities of the bank are overseen by the coordinator (first author). During the first year of operations, only 34 bone allografts were received in the bank but this number rose to a total of 88 allografts in the second year. This was a great feat and could be a result of awareness amongst orthopaedic surgeons and resident doctors.

Currently, living patients are the only donor source available in our bank. We had no age limitation for donors in as much as the inclusion and exclusion criteria are met. Our experience with bone allograft procurement was not far from what most bone bank facilities experienced with most of their bone allografts gotten from femoral heads of living patients [6-8], [12,13]. Most of the femoral head donors in our bank were elderly females who had hemiarthroplasty on account of neck of femur fracture. The female predominance of the donors could be explained by the high incidence of osteoporotic femur neck fractures in that gender. This finding is in variance with donor bio-demographics of a bone bank that reported male predominance [14].

To reduce the risk of disease transmission, we screened prospective donors of viral diseases such as presence of hepatitis B surface antigen, hepatitis C and HIV. All donors were seronegative as at the time of screening. Serological testing of the donors as per the pre-operative work-up included C-reactive protein, complete blood count and Erythrocyte sedimentation rate (ESR). Diverse graft sterilization methods are available to reduce transmission of infectious agents when processing human tissues and the selection of one over the other is predicated on the tissue bank protocol. Pruss *et al*. [15] decontaminated grafts with peracetic acid and reported that no viable micro-organisms were detected in any of the peracetic acid- ethanol (PES)-treated test cuboids. They concluded that PES-treatment of bone allograft is a reliable sterilization method. Terminal sterilization of bone allograft with gamma irradiation is also a well-known method and this is the preferred method in our bank. Our practice is similar to what was adopted in Tata Memorial Hospital, India where 25 kGy was also used for terminal sterilization [12]. Nguyen *et al*. [16] however studied the effects of gamma irradiation on allograft biology and biomechanics. They observed that the effect of irradiation is dose-dependent but there was no consensus on an optimum dose of radiation due to a wide range of confounding variables such as age, sex, size of graft. Low dose 25 kGy seems to be safe but there is a need for global studies of bone allograft qualities such as sterility, mechanical properties and biological functions after gamma irradiation. The rate of radiographic union or fusion of irradiated bone allograft implanted is currently being studied in our bank and we hope to publish our findings once the study is concluded. The reported allograft-related infection rate from studies ranged from 1.3% to 12.2% [17,18] whereas there was no incidence of clinical infection in this present study with only 28 allografts implanted. This could be possibly due to small sample size studied and could be due to irradiation of the grafts which were not done in the studies with higher infection rates. The donor selection criteria, recipient´s immune status, aseptic technique during harvest and implantation as well as graft processing techniques are critical in the prevention of allograft-related infection. Bone allografts from patients with infection or malignancy were discarded and no graft was harvested from fresh dead individuals. These were some of the ways the risk of disease transmission was prevented at our bank. Due to possible seroconversion of HIV, some bone banks adopted re-screening program for living donors at 3-6 months and would only certify grafts from seronegative donors fit for distribution [3,6,7,19,20]. We adopted gamma irradiation of the graft without a second HIV testing of the donors because it is believed that gamma rays will destroy all forms of micro-organisms (including virus and prions) within the contaminated grafts. The storage of irradiated graft in ultra-low freezer of -80°C helps to suppress the growth of micro-organisms and preserve the graft for about 5 years. We also maintained cold chain during graft transport and delivery within and outside the hospital. Accurate documentation and indexing of graft are very important in recording excellent performance in bone bank activities. Our bone bank clerk gives a registration code and creates a file for each donated graft. This is the standard practice in all bone banks to prevent technical errors that could lead to adverse outcomes. The allografts were utilized for various orthopaedic conditions with acetabular reconstruction being the most common indication. This finding is similar to reports from Nielsen *et al*. [21] but contrary to what was reported by Tomak *et al*. where most of the recipients were tumor cases [22].

Our challenges are also similar to Ward *et al*. recent systematic review of barriers to sustainability of bone banking programs in low- and middle-income countries. They identified lack of regulation, low donor rates, and insufficient training and staffing as primary barriers [23]. The low donor rate of long bones in instances of traumatic amputation can be ascribed to ignorance, cultural and religious beliefs on the part of the patients and relatives. Similarly, Stepanovic *et al*. [24] reported inadequate institutional support and donor disinterest to participate in bone banking as one of their challenges for quality control of activities of bone bank in a developing country. The low patronage among surgeons in our locality was partly due to lack of awareness of existence of bone bank services and partly due to infection risk and possible poor osteointegration of allograft. The patronage can be improved by constant awareness via seminars, paper presentation and publication of our processing technique and outcomes to allay their fears and instill confidence in the bone bank services. The success of the bone bank requires regular funding, training and medical supervision for exclusion of inappropriate donors, processing, indexing, storage and distribution of the graft until implantation into the recipient [25]. A regular audit of the bone bank will also improve the services rendered.

## Conclusion

This is a preliminary report on the running of the first bone bank facility in Nigeria. Bones from living donors are what is currently being processed in our bank. Our preferred technique of bone allograft processing, storage and distribution is safe. However, some of the challenges can be surmounted by funding, re-training of staffs and creating awareness amongst the populace and surgeons of the availability and safety of bone allografts.

### What is known about this topic

- There are established bone banks in some parts of Africa with different techniques of bone allograft processing.

### What this study adds

- To highlight the processing techniques and challenges encountered during the first 2 years of operation of the first bone bank facility in Nigeria.
